# HIV, cyclophilins and nuclear entry: going under the radar

**DOI:** 10.1186/1742-4690-8-S2-O7

**Published:** 2011-10-03

**Authors:** Greg Towers

**Affiliations:** 1 University College London, UK

## 

Lentiviruses such as HIV-1 traverse nuclear pore complexes (NPC) and infect terminally differentiated non-dividing cells, but how they do this is unclear. Here we define a pathway involving direct interaction between HIV-1 capsid (CA) and cytoplasmicCyclophilinA (CypA), and the nuclear pore cyclophilin Nup358/RanBP2. We show that these interactionsare essential for integration targeting and replication in primary human macrophages. In contrast to CypA Nup358 cyclophilin is insensitive to inhibition with cyclosporine. Inhibition of CypAusing cyclosporine or depletion of CypA levels by RNAi forces HIV-1 to enter the nucleus independently of Nup358 and the nuclear basket protein Nup153, suggesting that CypA regulates the choice of the nuclear import machinery that is engaged by the virus. Substitutions in capsid modulating CypA/Nup358 interactions switch integration into higher or lower gene density regions. HIV-1 cyclophilin-binding mutants CA G89V, P90A, or chimaeric HIV-1 containing SIVmac CA integrate in genomic areas of high gene density and activity, phenocopying integration of wild type virus in the presence of cyclosporine. In contrast, HIV-1 CA mutants that neither use Nup358 nor transportin 3 (CA N74D, N57A) integrate in genomic areas of low gene density and activity. Importantly, both groups of CA mutants are impaired in replication in HelLa cells and human monocyte derived macrophages. Our findings illustrate how HIV-1 engagescyclophilins to select a nuclear entry pathway required for integration into preferred genomic loci important for optimal proviral gene expression and replication and provide insight into lentiviral conservation of cyclophilin interactions.

